# Biliary pancreatic diversion and laparoscopic adjustable gastric banding in morbid obesity: their long-term effects on metabolic syndrome and on cardiovascular parameters

**DOI:** 10.1186/1475-2840-8-37

**Published:** 2009-07-20

**Authors:** Antonio E Pontiroli, Marco Laneri, Annamaria Veronelli, Francesca Frigè, Giancarlo Micheletto, Franco Folli, Gianfranco Adami, Nicola Scopinaro

**Affiliations:** 1 Dipartimento di Medicina, Chirurgia e Odontoiatria, Università degli Studi di Milano, Ospedale San Paolo, Milano, Italy; 2 Dipartimento di Scienze Chirurgiche, Milano, Italy; 3 Diabetes Division, Department of Medicine, University of Texas Health Science Center at San Antonio, San Antonio, Texas, USA; 4 Dipartimento di Discipline Chirurgiche, Università di Genova, Genova, Italy

## Abstract

**Background:**

Bariatric surgery is able to improve glucose and lipid metabolism, and cardiovascular function in morbid obesity. Aim of this study was to compare the long-term effects of malabsorptive (biliary pancreatic diversion, BPD), and restrictive (laparoscopic gastric banding, LAGB) procedures on metabolic and cardiovascular parameters, as well as on metabolic syndrome in morbidly obese patients.

**Methods:**

170 patients studied between 1989 and 2001 were called back after a mean period of 65 months. 138 patients undergoing BPD (n = 23) or LAGB (n = 78), and control patients (refusing surgery and treated with diet, n = 37) were analysed for body mass index (BMI), blood glucose, cholesterol, and triglycerides, blood pressure, heart rate, and ECG indexes (QTc, Cornell voltage-duration product, and rate-pressure-product).

**Results:**

After a mean 65 months period, surgery was more effective than diet on all items under evaluation; diabetes, hypertension, and metabolic syndrome disappeared more in surgery than in control patients, and new cases appeared only in controls. BPD was more effective than LAGB on BMI, on almost all cardiovascular parameters, and on cholesterol, not on triglyceride and blood glucose. Disappearance of diabetes, hypertension, and metabolic syndrome was similar with BPD and with LAGB, and no new cases were observed.

**Conclusion:**

These data indicate that BPD, likely due to a greater BMI decrease, is more effective than LAGB in improving cardiovascular parameters, and similar to LAGB on metabolic parameters, in obese patients. The greater effect on cholesterol levels is probably due to the different mechanism of action.

## Background

Obesity is often accompanied by diabetes, hyperlipidemia, arterial hypertension [1]; all are known risk factors for cardiovascular (CV) diseases, i.e. accelerated atherosclerosis [2] and congestive heart failure [CHF, [3]]. Insulin resistance [4], subclinical inflammation [5], sympathetic overactivity [6], and endothelial dysfunction [7], are present and often coexist in the same individual [8]. These abnormalities are risk factors for the development of left ventricular hypertrophy [LVH, [9,10]], which is frequently detected in severe obesity, and is an alleged risk factor for CHF [3,10]. The rate pressure product (RPP, heart rate × systolic BP) is a correlate of myocardial oxygen consumption, and hence of work load of the heart [11], is raised in obesity [11-13]; RPP is considered a determinant of cardiovascular risk, since its increase precedes ischemic events [11].

Bariatric surgery improves glucose and lipid metabolism [14,15] and attenuates endothelial dysfunction [16] and sympathetic overactivity [17]. These changes are inter-related, since reduction of sympathetic overactivity, improvement of endothelial dysfunction, and decrease of insulin resistance correlate each other, and with decreases of body weight and of visceral fat [18]. In addition, bariatric surgery has been shown to prevent arterial hypertension [19] and diabetes mellitus [19-21].

Bariatric surgery has been reported to reduce LVM indices in a number of studies, and has beneficial effects on virtually all sections of electrocardiogram (ECG)[22]. It is possible that weight loss is effective in reducing LVM if accompanied by decrease of blood pressure [17]. LVM correlates with circulating leptin levels [23], and with insulin resistance [24], and decreases of LVM and of leptin levels are correlated [23] in obese normotensive subjects.

The amount of weight loss is greater with malabsorptive than with restrictive bariatric procedures [14,15,25]. Only few studies have directly compared different techniques [21,26-28], and were usually of short duration, and without control patients, i.e. patients not undergoing surgery [except for ref. [21]]. The aim of this study was to compare the long-term effects of malabsorptive (biliary pancreatic diversion, BPD], and restrictive (laparoscopic gastric banding, LAGB) procedures on metabolic and cardiovascular parameters, and on metabolic syndrome, in morbidly obese patients.

## Methods

Laparoscopic gastric banding (LAGB) and biliary pancreatic diversion (BPD) are routinely performed at participating Institutions, following the indications and guidelines of NIH [29]; procedures have been approved by local ethics committees; patients undergo preliminary clinical work-up including psychological and psychiatric evaluation [14].

The protocol of this study, i.e. ad-hoc call-back of patients for selected determinations, was specifically approved by local ethics committees, and all participating patients gave written informed consent. Patients undergoing surgery between 1989 and 2001 were considered in this study; all patients agreeing to come back to Institutions were considered; they were interviewed about current therapies, and underwent anthropometric evaluation, ECG recording, and heart rate and blood pressure measurement, fasting blood sampling for glucose and lipid determination. Patients who refused surgery and agreed to be followed-up (controls), were also considered. They were treated with diet [14]. Patients with atrial fibrillation or left bundle branch block were excluded, as well as patients receiving drugs known to interfere with electrophysiological ECG conduction. Therefore, a total number of 32 patients could not be included in the study because of the above clinical conditions or because basal ECG paper tracings had vanished and become unreadable; these patients were not different from the remaining 138 patients as to baseline characteristics or to clinical and metabolic follow-up. Blood sampling [14,15] and ECG tracing in all patients was carried out at rest, after an overnight fast. On the same occasion, arterial blood pressure was evaluated by the same physician, using the same sphygmomanometer with an appropriate cuff.

### Outcome measures

Arterial hypertension was diagnosed when systolic/diastolic blood pressure was ≥ 140/90 mmHg, or when subjects were under antihypertensive treatment. Diabetes mellitus was diagnosed when fasting blood glucose levels were > 126 mg/dl, or when subjects were under antihyperglycemic treatment (metformin). Rate pressure product (RPP, mmHg × bpm × 10^-2^) was calculated, and was used as an index of myocardial oxygen consumption and hence of work load of the heart [11]. Diagnosis of metabolic syndrome was established according to the ATP III criteria [30], and included three or more components.

### Interviews and anthropometry

Patients were interviewed as to current and past therapies, and were measured for height and weight, to calculate body mass index, as already reported [14,15].

### ECG: QTc interval, Cornell voltage-duration product

All individual ECGs performed at baseline and at follow-up were compared by one person (ML). QT intervals were measured manually from the onset of the interval between Q and S waves of the electrocardiogram to the end of the T wave on the isoelectric baseline, and corrected according to Bazett's formula (QTc = QT/√RR) [31]. When a T wave could not be reliably determined, the lead was excluded from analysis [32]. QT interval was measured in at least 10 leads in each subject. Through ECG reading (paper running at 25 mm/s, 1 mm/mV), the Cornell voltage-duration product was calculated: [(RaVL+SV3) × QRS] in mm.ms, with an adjustment of 6 mm in women. These composite ECG criteria detect LVH with about 95% specificity in healthy and in hypertensive subjects [33-35], and this approach allows direct evaluation of LVM [36-38].

### Statistical analysis

Inter-group comparisons were performed by one way analysis of variance (ANOVA) followed by Scheffè's multiple comparison test. Intra-group comparisons were performed by two-tailed Student's t test for paired samples. Since a normal distribution of data was not assured, non-parametric tests (Mann-Whitney and Wilcoxon test) were also used. Absolute frequencies and changes of comorbidities were compared by χ^2 ^test. Pairwise correlations were computed between changes of clinical conditions (independent variables) and change of Cornell voltage-duration product, and of RPP (dependent variables). Next, a multivariate regression analysis was carried out to assess the role of independent variables significant at univariate analysis (plus age and sex) in change of clinical conditions such as diabetes mellitus, arterial hypertension, and metabolic syndrome, and changes of Cornell voltage-duration product, and of RPP; we reported significance of the whole model, plus F and p of variables statistically significant at multivariate regression analysis. *p *levels < 0.05 were considered statistically significant.

## Results

**Figure 1:**
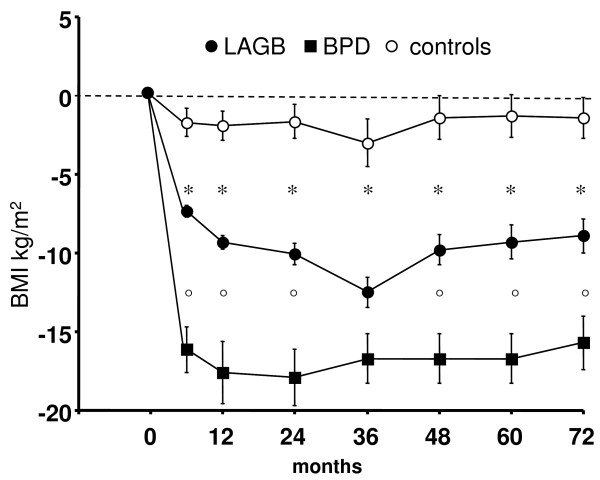
**Decrease of body mass index (BMI, kg/m^2^) in patients undergoing BPD, LAGB, and in control patients**. Means ± SE. at all times intervals, BMI decrease was significantly greater with BPD than with LAGB (°, p < 0.05 or less), and with LAGB than in controls (*, p < 0.05 or less).

Figure 1 shows decrease of BMI in patients undergoing surgery and in control patients: weight loss was greater with BPD than with LAGB, and greater with LAGB than in controls, at virtually all time intervals. Table 1 shows that surgery, whether BPD or LAGB, was significantly superior to traditional treatment on all parameters considered. Diabetes mellitus and arterial hypertension disappeared in a few surgery patients, and appeared in a few control patients. Patients with type 2 diabetes mellitus differed from other patients only for older age and for greater decrease of blood glucose levels. Table 2 shows the effects of BPD and of LAGB on metabolic and cardiovascular parameters. BPD was statistically more effective on weight loss, on decrease of systolic and diastolic blood pressure, and on decrease of Cornell voltage-duration product and of RPP, and hence on reduction of cases of LVH. BPD, at difference from LAGB was also effective in decreasing cholesterol levels, while the effect on blood glucose and on triglycerides, and on disappearance of metabolic syndrome, was not different.

**Table 1:** Clinical, ECG, and metabolic details of patients in the study (controls and patients undergoing surgery).

	CONTROLS			SURGERY		
	BEFORE	AFTER	CHANGE	BEFORE	AFTER	CHANGE
N (men, women)	37 (5/32)			101 (17/84)		
Age (y)	46.5 ± 2.07			44.8 ± 0.98		
Interval (mos)	59.3 ± 4.44			65.4 ± 4.65		
BMI (kg/m^2^)	43.8 ± 1.18	43.5 ± 1.54	+0.3 ± 0.81	45.7 ± 0.67	34.2 ± 0.59 #	-11.1 ± 0.71 #
Systolic BP (mmHg)	133.5 ± 2.39	140.5 ± 3.12	+7.0 ± 3.82	136.4 ± 1.65	127.1 ± 1.48 #	-9.4 ± 1.95 #
Diastolic BP (mmHg)	83.7 ± 1.52	89.4 ± 1.91	+5.8 ± 2.46	85.9 ± 1.34	81.1 ± 1.01 #	-4.7 ± 1.53 #
Hypertension (y/n)	14/23	21/16	+7 (-3, +10)	40/61	20/81 #	-20 (-23, +3) §
Heart rate (bpm)	74.9 ± 1.83	74.2 ± 1.84	-0.6 ± 2.31	80.4 ± 1.29 *	67.5 ± 0.98 #	-12.9 ± 1.24 #
QTc (msec)	412.8 ± 4.62	410.7 ± 4.88	-2.1 ± 5.41	412.4 ± 2.99	398.7 ± 2.81 *	-13.7 ± 3.03 *
Cornell-voltage (mm.ms)	1487.3 ± 84.75	1434.6 ± 81.64	-52.7 ± 62.43	1561.1 ± 57.77	1461.3 ± 57.39	-106.1 ± 59.44
RPP	99.7 ± 2.99	104.7 ± 3.91	+5.6 ± 4.43	109.7 ± 2.39 *	86.2 ± 1.58 #	-23.6 ± 2.42 #
LVH (y/n) °	8/29	5/32	-3 (+2, -5)	31/70	28/73	-3 (+9, -12)
Cholesterol (mg/dl)	195.4 ± 8.66	198.9 ± 6.88	+2.1 ± 5.17	203.5 ± 4.47	183.5 ± 5.82	-20.4 ± 6.55 *
Triglycerides (mg/dl)	114.1 ± 8.57	123.9 ± 8.96	+9.4 ± 8.04	162.4 ± 17.13	100.3 ± 5.84 *	-64.1 ± 17.14 *
Blood glucose (mg/dl)	117.7 ± 8.74	124.6 ± 9.63	+6.9 ± 6.77	106.2 ± 3.58	91.1 ± 1.91 #	-15.1 ± 3.18 **
Diabetes mellitus (y/n)	9/28	11/26	+2	14/87	4/97 #	-10 *
Metabolic Syndrome (y/n)	15/22	13/24	-2 (-6, +4)	30/71	11/90 **	-19 *

Means ± SE or absolute frequencies

° as defined by ECG criteria

* = p < 0.05; ** = p < 0.01; # = p < 0.001 vs corresponding values of controls

**Table 2:** Clinical, ECG, and metabolic details of patients undergoing biliary-pancreatic-diversion (BPD) and laparoscopic gastric banding (LAGB).

	BPD			LAGB		
	BEFORE	AFTER	CHANGE	BEFORE	AFTER	CHANGE
N (men, women)	23 (7/16)			78 (10/68)		
Age (y)	45.3 ± 1.78			44.2 ± 1.16		
BMI (kg/m^2^)	48.6 ± 1.45	30.1 ± 1.26	-18.5 ± 1.43	44.8 ± 0.74 *	35.4 ± 0.61 #	-9.5 ± 0.66 #
Systolic BP (mmHg)	148.3 ± 4.98	121.7 ± 2.86	-26.5 ± 5.16	132.9 ± 1.35 #	128.6 ± 1.69 *	-4.3 ± 1.61 #
Diastolic BP (mmHg)	97.8 ± 3.81	76.3 ± 1.92	-21.5 ± 3.35	82.4 ± 1.04 #	82.6 ± 1.12 *	+0.1 ± 1.26 #
Hypertension (y/n)	12/11	1/22	-11	28/50	19/59	-9 (-12, + 3)
Heart rate (bpm)	82.4 ± 3.63	69.8 ± 2.22	-12.6 ± 2.98	79.8 ± 1.31	66.8 ± 1.09	-13.0 ± 1.36
QTc (msec)	403.5 ± 5.44	388.9 ± 6.27	-14.6 ± 5.83	415.1 ± 3.48	401.6 ± 3.09	-13.5 ± 3.54
Cornell-voltage (mm.ms)	1612.6 ± 155.48	1249.6 ± 144.63	-391.4 ± 172.87	1545.9 ± 59.69	1523.8 ± 59.62	-22.2 ± 54.97 **
RPP	122.9 ± 7.79	85.4 ± 3.84	-37.5 ± 6.96	105.9 ± 1.92 *	86.4 ± 1.71	-19.5 ± 2.19 **
LVH (y/n) °	8/15	2/21	-6	23/55	26/52	+3 (-6, +9) *
Cholesterol (mg/dl)	210.1 ± 9.78	133.8 ± 6.43	-76.3 ± 11.48	200.5 ± 4.75	205.8 ± 5.26 #	+5.3 ± 3.82 #
Triglycerides (mg/dl)	190.4 ± 45.58	83.3 ± 7.02	-107.0 ± 48.33	149.6 ± 12.32	108.1 ± 7.69 *	-41.4 ± 10.31
Blood glucose (mg/dl)	98.8 ± 7.66	82.1 ± 2.13	-16.7 ± 6.94	108.4 ± 4.04	93.7 ± 2.31 **	-14.7 ± 3.49
Diabetes mellitus (y/n)	2/21	0/23	-2	12/66	4/74	-8
Metabolic Syndrome (y/n)	5/18	0/23	-5	25/53	11/67	-14

Means ± SE or absolute frequencies.

° as defined by ECG criteria

* = p < 0.05; ** = p < 0.01; # = p < 0.001 vs corresponding values of BPD

In surgery patients, decrease of systolic and diastolic blood pressure, of Cornell voltage-duration product, of RPP correlated with decrease of BMI. The decrease of cholesterol was greater with BPD than with LAGB, independent of initial BMI, and of the amount of weight loss (not shown), and therefore seems due to the kind of surgery rather than to the amount of weight loss.

At multiple regression analysis disappearance of diabetes mellitus was significantly associated with presence of diabetes at the beginning (model r = .559, F = 30.013, p = 0.0001); disappearance of arterial hypertension was associated (model r = .562, p = 0.0001) with presence of hypertension at the beginning (F = 24.415, p = 0.0001) and change of BMI (F = 10.138, p = 0.02); disappearance of metabolic syndrome was associated (model r = .763, p = 0.0001) with presence of metabolic syndrome at the beginning (F = 53.589, p = 0.0001) and with change of BMI (F = 6.739, p = 0.0115). Table 3 shows that change of Cornell voltage-duration product correlated with initial Cornell voltage-duration product, with change of blood pressure and of BMI; at multiple regression analysis, the model accounted for r = .635, and only initial Cornell voltage-duration product, and change of systolic BP correlated with change of Cornell voltage-duration product. Table 3 also shows that change of RPP correlated with initial RPP, HR and blood pressure, and with change of systolic blood pressure, of heart rate, and of BMI; at multiple regression analysis, the model accounted for r = .995, and initial RPP, HR and BP, and change of HR and of systolic BP correlated with change of RPP.

**Table 3:** Linear and multiple regression

	Linear Regression	Multiple Regression
A		
Δ Cornell Voltage Product		Model r = .635,
Δ BMI	.204, 0.0406	
Cornell voltage product initial	.516, 0.0001	F = 45.471, 0.0001
Δ Systolic BP	.282, 0.0001	F = 9.616, 0.01
Δ Diastolic BP	.235, 0.0001	
		
B		
Δ RPP		Model r = .989,
Δ BMI	.297, 0.0025	
RPP initial	.786, 0.0001	F = 158.97, 0.0001
Systolic BP	.491, 0.0001	F = 93.45, 0.0001
Diastolic BP	.387, 0.0001	
HR	.616, 0.0001	F = 110.42, 0.0001
Δ HR	.754, 0.0001	F = 1721.69, 0.0001
Δ Systolic BP	.685, 0.0001	F = 1060.15, 0.0001
Δ Diastolic BP	.489, 0.0001	

A. On the left: correlations (linear regression) between changes of Cornell voltage product (dependent variable) and changes of clinical variables (independent variables): r and p are indicated. On the right, multiple regression model, partial F and p of independent variables statistically significant. B. On the left: correlations (linear regression) between changes of RPP (dependent variable) and changes of clinical variables (independent variables): r and p are indicated. On the right, multiple regression model, partial F and p of independent variables statistically significant.

## Discussion

BPD is known to induce a greater weight loss than LAGB, independently of initial BMI; this information comes from a meta-analysis [25] and from two comparative studies [27,28]. This finding was confirmed in the present study. Surgery was clearly more effective than control treatment, as already reported in the only study including control patients [21]. The pattern of BMI depicted in Figure 1 indicates that our findings were representative of what happens with the two surgical procedures. In agreement with two small studies [27,28], the effect on metabolic variables, and the disappearance of diabetes mellitus (patients were treated with metformin, when required) were not different for the 2 procedures. Also disappearance of metabolic syndrome was not different. As to metabolic effects, the only difference was in cholesterol decrease, which appears more due to the technique than to amount of weight loss. We have other data [39] showing that another malabsorptive technique (Bilio-Intestinal-By-Pass) is more effective than LAGB in reducing cholesterol levels. The cholesterol level reduction that we and others have reported is a quite dramatic phenomenon and is likely due to the major reduction in bile acid re-absorption in the intestine and possibly to altered regulation of the feedback mechanisms controlled by nuclear protein such as LXR, FXR and PPAR; these transcriptional factors are involved in bile acid and cholesterol metabolism, occurring in patients undergoing BPD (which causes malabsorption and also reduced bile re-absorption), but not LAGB (a purely restrictive bariatric procedure)[14,18,19,40-42]. It is also possible that reduced gastric volume and reduced production of gastric lipase, as well as reduced secretion of colecystokinin (that physiologically stimulates digestive enzyme secretion such as lipases and proteases) might result in a marked decrease in the hydrolysis of triacylglycerols, with a reduction of the absorption of free fatty acids [43].

Some cardiovascular parameters were more influenced by BPD than by LAGB, such as diastolic and systolic blood pressure, Cornell voltage-duration product and RPP. It seems that this was generally due to a greater effect on BMI, although a direct role of BMI in multiple regression analysis was not evident.

Bariatric surgery is known to improve all sections of ECG [22]; ECG has been used as a method of measuring LVM (Cornell voltage-duration product) and of assessing work load [RPP, [11,33-38]]. In this study we found that BPD is superior to LAGB on these parameters. The fact that change of Cornell voltage-duration product is dependent first at all on its initial value fully agrees with the findings that decrease of LVM (echocardiographic measures) is dependent first at all with initial LVM [22]; in a previous paper we have shown that Cornell voltage-duration product and echocardiographic measures similarly describe decrease of LVM after weight loss, correlated with decrease of leptin levels [23]. Obesity is often accompanied, especially when of long duration, by increase of LVM [22]; probably, when obesity is untreated, the natural trend is of a progressive increase of LVM. Weight loss can reduce LVM [13,17,22], especially if accompanied by reduction of blood pressure [17]. In this study we found a significant reduction of Cornell voltage-duration product together with a significant decrease of both diastolic and systolic blood pressure. This finding corroborates a previous study from this group showing that weight loss alone is not sufficient to allow decrease of LVM [17].

HR and QTc are assumed as an index of sympathetic overactivity [17]; their decrease after weight loss is well in agreement with a previous study showing enhanced parasympathetic activity after diet-induced weight loss [44]. Rate pressure product, a correlate of myocardial oxygen consumption, and hence of work load of the heart [11,12], decreased with weight loss. This finding has never been published before. Decrease of RPP correlated with initial RPP as well as with decrease of BMI, and of diastolic BP. Given the value of RPP and of heart rate as determinants of cardiovascular risk [11,45,46], these data indicate that both weight loss and decrease of blood pressure are of importance in reducing the overall cardiovascular risk in morbid obesity.

## Conclusion

These data indicate that BPD, likely due to a greater BMI decrease, is more effective than LAGB in improving cardiovascular parameters, and similar to LAGB on metabolic parameters, in obese patients. The greater effect on cholesterol levels is probably due to the different mechanism of action.

## Abbreviations

**ANOVA**: analysis of variance; **ATP III**: adult treatment panel III; **BMI**: body mass index; **BP**: blood pressure; **BPD**: biliopancreatic diversion; **CHF**: congestive heart failure; **CV**: cardiovascular; **ECG**: electrocardiography; **FXR**: farnesoid X-receptor; **HR**: heart rate; **LAGB**: laparoscopic adjustable gastric banding; **LVH**: left ventricular hypertrophy; **LVM**: left ventricular mass; **LXR**: liver X-receptor; **PPAR**: peroxisome proliferator activated receptor; **QTc**: corrected QT interval; **RPP**: rate pressure product.

## Competing interests

The authors declare that they have no competing interests.

## Authors' contributions

AEP, FF, GM, GA, and NS designed the study protocol. GM, AV, ML, FF were in charge of evaluating patients, organizing databases, performing statistical analysis. GM, GA, and NS were the surgeons performing all surgical procedures. All authors substantially contributed to writing the manuscript. All authors read and approved the final manuscript.
